# The biology and ecology of the Pacific sharpnose shark *Rhizoprionodon longurio*

**DOI:** 10.1002/ece3.10600

**Published:** 2023-10-11

**Authors:** Joel H. Gayford, Darren A. Whitehead

**Affiliations:** ^1^ Department of Life Sciences Silwood Park Campus, Imperial College London London UK; ^2^ Shark Measurements London UK; ^3^ Investigación Tiburones México A.C La Paz Mexico

**Keywords:** carcharhinid, carcharhiniformes, climate change, elasmobranch, overfishing, resilience

## Abstract

Amidst global declines in elasmobranch populations resulting predominantly from overfishing, the need to gather data regarding shark ecology is greater than ever. Many species remain data deficient or at risk of going extinct before sufficient conservation measures can be applied. In this review, we summarise existing knowledge regarding the biology and ecology of the Pacific sharpnose shark *Rhizoprionodon longurio* (Jordan & Hilbert, 1882), a small‐bodied carcharhinid shark found in coastal waters of the Eastern Tropical Pacific Ocean that is of both commercial and ecological importance. We compare ecological parameters of this species with its closest extant relatives and identify major knowledge gaps and avenues for future research. In particular, additional studies investigating the behavioural and sensory ecology, as well as potential migratory patterns of the species are needed. Such studies will not only improve our understanding of *R. longurio*, but provide insight into the extent to which the numerous studies performed on a close relative—*Rhizoprionodon terraenovae*—provide an accurate representation of the biology and ecology of Rhizoprionodon and carcharhinids more generally.

## Graphical abstract

Despite its ecological and commercial importance, the Pacific sharpnose shark (*Rhizoprionodon longurio*) is understudied compared to other members of its genus. Here, we summarise existing knowledge regarding the biology and ecology of the species and suggest areas for future research.

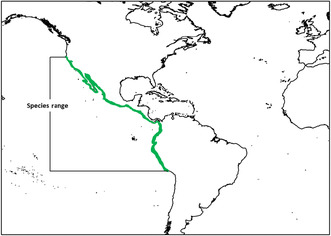

## 1 OVERVIEW

Sharks and their relatives are in global decline (Pacoureau et al., 2021), with over one third of all species vulnerable to extinction (Dulvy et al., 2021). Overfishing, climate change and habitat destruction all represent major threats to elasmobranch diversity (Jorgensen et al., 2022; Rosa et al., 2017; Ward‐Paige & Worm, 2017), compounded by the supposed innate vulnerability of slowly reproducing *k strategists* to extinction (Cortés, 2000). For these reasons, studies are urgently required to gather information regarding the biology, ecological functions and ecology of elasmobranchs to facilitate appropriate conservation measures. Given the difficulty in maintaining laboratory populations of most shark species and the animal ethics involved with husbandry care, studies focussing on wild populations are vital. The great taxonomic diversity of elasmobranchs (Compagno, 1984) means that a most species remain poorly understood (Jorgensen et al., 2022). Identifying accessible ‘model taxa’ that are biologically and ecologically representative of other, less accessible taxa should be a priority of contemporary shark research alongside efforts to categorise the population‐level health of as many species as possible, ensuring that prioritisation of research effort can be made based on relative vulnerability to extinction.

*Rhizoprionodon* is a genus of small‐bodied carcharhiniform sharks consisting of seven extant species distributed globally in tropical and temperate coastal waters (Compagno, 1984). Despite their conserved external morphology (Pinhal et al., 2012), *Rhizoprionodon* species exhibit clear differences in ecology and biology (Corro‐Espinosa et al., 2011; Springer, 1964). *R. terraenovae* is by far the most intensively studied of the extant *Rhizoprionodon* species; however, the extent to which the characteristics of *R. terraenovae* are representative of the genus as a whole has not been assessed. The Pacific sharpnose shark *R. longurio* (Jordan & Hilbert, 1882) is a comparatively understudied species found in shallow coastal waters of the Eastern Tropical Pacific Ocean from southern California to Peru (Compagno, 1984). Until recently, *R. longurio* was considered data deficient (Pinhal et al., 2012) and only in recent years has literature focussing on the species increased substantially. *R. longurio* is significantly more vulnerable to extinction than *R. terraenovae* on the basis of current IUCN estimates (Carlson et al., 2021; Pollom et al., 2020) and the species is subject to greater fishing pressure than *R. terraenovae* over much of its range (Corro‐Espinosa et al., 2011; Pérez‐Jiménez et al., 2015). For these reasons, improving our understanding of *R. longurio* biology and ecology is vital so that we may provide biologically informed conservation measures and maintain ecological function.

In this article, we summarise existing knowledge regarding the distribution, spatial ecology, growth, reproduction, trophic ecology and behaviour of *R. longurio* and provide comparisons to other members of the *Rhizoprionodon* genus. It has been 18 years since the last such review (Márquez‐Farias et al., 2005), and during this time several studies have addressed previously unknown aspects of *R. longurio* ecology. As well as synthesising current knowledge, we comment on major knowledge gaps that constrain our understanding of the species and how they might be overcome.

## 2 TAXONOMY AND EVOLUTION

*Rhizoprionodon longurio* is a small‐bodied Carcharhiniform shark belonging to the genus *Rhizoprionodon* (Compagno, 1984). This genus is thought to have diverged approximately 53 million years ago (Carrier et al., 2012). As a relatively recent radiation, the genus *Rhizoprionodon* could prove a valuable case study for investigating the forces driving speciation in sharks. Several abiotic and biotic factors can drive such radiations (Simões et al., 2016; Solórzano et al., 2020); however, it is particularly challenging to identify these in ancient radiations where much subsequent evolution has occurred and patterns of genetic diversity can mask signatures of historical demographic events (Dudgeon et al., 2020; Richards et al., 2019). As well as the relatively recent nature of the *Rhizoprionodon* radiation, the geographical distribution of its constituent taxa warrants mention. Genera such as *Hemiscyllium*, the speciation and biogeography of which has been focus of recent studies (Dudgeon et al., 2020) exhibit relatively restricted geographical distribution, whereas *Rhizoprionodon* taxa are found worldwide in the coastal waters of the Pacific, Atlantic and Indian oceans (Pinhal et al., 2012). Regardless, as of yet no studies have considered biogeography or speciation of the genus *Rhizoprionodon* specifically.

*Rhizoprionodon* taxa exhibit the ‘typical’ carcharhiniform body form (Sternes & Shimada, 2020; Thomson & Simanek, 1977); however, they differ from other carcharhinid sharks through a combination of morphological characters including enlarged hyomandibular pores and well‐developed labial furrows (Springer, 1964), as well as the characteristic elongated snout from which the common name ‘sharpnose shark’ arises (Pinhal et al., 2012). The seven extant *Rhizoprionodon* species exhibit relatively conservative external morphology (Pinhal et al., 2012), however have previously been subdivided into two groups on the basis of features relating to vertebral centra morphology (Springer, 1964). Interestingly this taxonomic relationship is also recovered by molecular phylogenies (Naylor et al., 2012), through which *Rhizoprionodon lalandii* (another highly understudied species) can be defined as the sister taxon of *R. longurio*.

*R. longurio* can also be distinguished from other *Rhizoprionodon* species morphologically since it has a relatively high tooth count, particularly elongated rostrum, characteristic upper labial furrow length and an absence of gyandric heterodonty (sexual dimorphism in dentition) (Springer, 1964). No existing studies expand on the potential for sexual dimorphism in *R. longurio* despite the abundance of sex‐based morphological differences in other sharks and their potentially significant ecological and behavioural implications (Ritter & Amin, 2019; Whitehead et al., 2022). A lack of dental sexual dimorphism may suggest that sexual conflict is relatively weak compared with other elasmobranchs, however the absence of dental sexual dimorphism alone is insufficient to confirm this (Gayford, 2023). The morphological characteristics of *R. longurio* are relatively well understood compared with other aspects of the species' ecology, and therefore there are no major knowledge gaps in this area that are specific to *R. longurio*. Future morphological studies should examine the potential for morphological differences across the species' range, the genetic/developmental architecture underlying morphology and finescale morphological differences between the sexes and through ontogeny.

## 3 DISTRIBUTION AND MOVEMENTS

**FIGURE 1:**
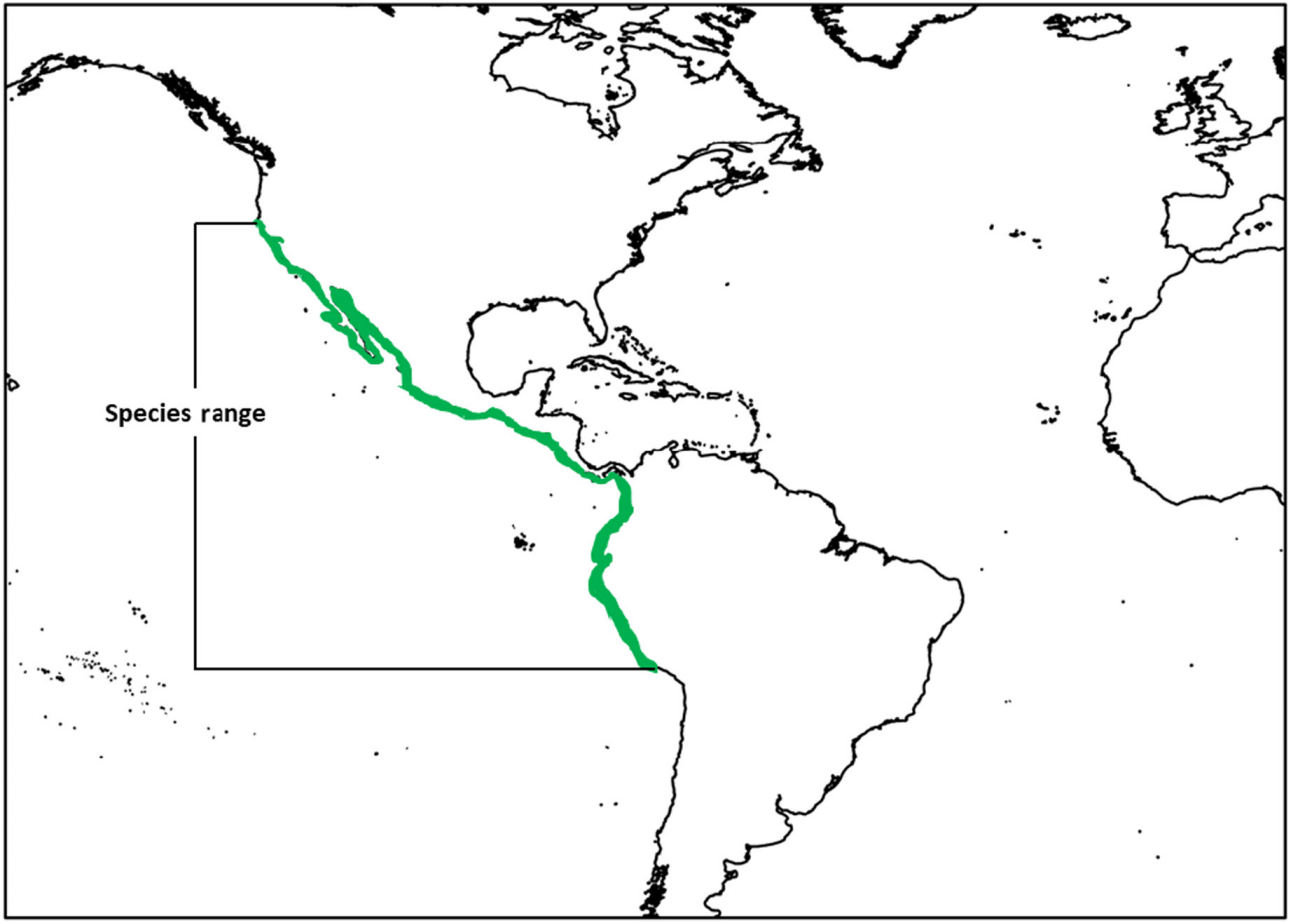
Map displaying the distribution of *Rhizoprionodon longurio* along the Western coast of The Americas, from Southern California to Perú.

Understanding shark distribution and movement patterns is critical to ensuring their protection (Hammerschlag et al., 2011; Schlaff et al., 2014), as well as their ecological functions and interactions with other taxa (Bird, 2017; Tickler, 2021). *R. longurio* is found in the coastal waters of the Eastern Tropical Pacific Ocean, between Southern California and Perú (Figure 1; Compagno, 1984). It is the sole *Rhizoprionodon* species to inhabit this region, and in fact the only *Rhizoprionodon* species to live in isolation from all other members of the genus throughout its range (Pinhal et al., 2012). *R. longurio* is a benthopelagic species found in inshore waters in association with sandy and/or muddy substrates (Compagno, 1984; Corro‐Espinosa et al., 2011; Márquez‐Farias et al., 2005). Their ecology is broadly similar to other *Rhizoprionodon* species (Carlson et al., 2008; Munroe et al., 2014), although unlike *R. taylori*, *R. longurio* is not known to use seagrass habitats. This may be due to trophic differences between these species or due to a higher prevalence of sea grass habitats in the Western Pacific than the Eastern Tropical Pacific (Munroe et al., 2014; Short et al., 2001). Until recently, the habitat usage of *R. longurio* was unknown, however recent studies suggest that juveniles may utilise nursery areas for foraging purposes and/or protection from potential predators (Trejo Ramírez, 2017). These are typically shallow, sheltered coastal regions including bays, mangroves and lagoons in which predation pressure is thought to be lower than the surrounding habitats (Heupel et al., 2007; Kinney & Simpfendorfer, 2009). Many carcharhiniform sharks, including some other *Rhizoprionodon* species are known to utilise nursery areas (Castro, 1993; Yokota & Lessa, 2006), and thus it is not surprising that *R. longurio* should engage in such behaviour. Intriguingly however, *R. terraenovae* is not thought to use shallow‐water nursery areas, and instead juveniles of this species frequently occupy deeper waters, in some cases for extensive periods of time (Carlson et al., 2008). Differences in the composition of respective communities, including differences in absolute predation pressure could be responsible for such a difference. It is also plausible that, at least during this early ontogenetic stage, *R. longurio* and *R. terraenovae* are occupying different ecological niches.

The most commonly stated observation regarding *R. longurio* movement in available literature regards a supposed northward seasonal migration in populations inhabiting the Gulf of California (Alatorre‐Ramirez et al., 2013; Corro‐Espinosa et al., 2011; Márquez‐Farias et al., 2005; Osuna‐Peralta et al., 2014). Unfortunately, little information is known about this putative migration, and such suggestions are based purely on fishing records and the tagging of a few individuals (Gayford et al., 2023; Kato & Carvallo, 1967). Seasonal variation in *Rhizoprionodon* catches is known from multiple species (Márquez‐Farias et al., 2005; Parsons & Hoffmayer, 2005); however, the nature and drivers of these putative migrations remain uncertain. Both climate (temperature/oxygen) and reproduction‐driven migrations are plausible (Parsons & Hoffmayer, 2005) as both are known to occur in other elasmobranch taxa (Kessel et al., 2014; Pratt & Carrier, 2001) and in many cases result in superficially similar patterns of dispersal and distribution (Parsons & Hoffmayer, 2005). In the case of *R. longurio*, migratory behaviour appears to be on a north–south axis (Alatorre‐Ramirez et al., 2013; Márquez‐Farias et al., 2005), however based on existing data inshore rather than latitudinal migration cannot be ruled out and future work should strive towards a greater understanding of these movements. Moreover, it is uncertain whether this migratory behaviour is observed across all size classes, or whether it is ontogenetically stratified (Gayford et al., 2023). *R. longurio* is often caught in gillnets (Márquez‐Farias et al., 2005) which due to their mesh size would likely not capture smaller juveniles, meaning that data regarding seasonal variations in *R. longurio* distribution to date are only applicable to larger animals. Acoustic telemetry studies have been conducted for some *Rhizoprionodon* taxa, providing valuable information about their habitat usage, residency and abundance (Carlson et al., 2008; Munroe et al., 2014; Reyier et al., 2023). Regrettably, data regarding the movements and spatial ecology of *R. longurio* are scarce. In addition to the uncertainties regarding migratory behaviour, nothing is known about diel and vertical movements of this species besides vague speculation based on trophic studies (Alatorre‐Ramirez et al., 2013; Osuna‐Peralta et al., 2014). Future studies utilising acoustic and satellite telemetry data will provide better understanding about the spatial ecology of *Rhizoprionodon* species, which given their ecological and commercial importance (Corro‐Espinosa et al., 2011; Pérez‐Jiménez et al., 2015) should be a priority. In particular, effort should be made to characterise habitat usage of *R. longurio* in the Southern parts of its range, which as of yet remains entirely unknown.

## 4 GROWTH AND REPRODUCTION

Accurate information regarding the growth parameters of elasmobranch taxa is crucial for ensuring proper management (Harry, 2018; Smart et al., 2016). Furthermore, an understanding of a species' reproductive biology assists in determining its vulnerability to population declines (Bejarano‐Álvarez et al., 2011; Carrier et al., 2004). Sharks are typically thought of as *k strategists* exhibiting slow growth rates and low fecundity (Cortés, 2000), and as such are often thought of as being particularly vulnerable to extirpation (Dulvy et al., 2021). The supposed dichotomy between *r* and *k strategists* in reality represents a continuum of life history strategies (Southwood et al., 1974) and there is considerable variation in fecundity and growth rates within Elasmobranchii (Cortés, 2000). *Rhizoprionodon* is a good example of this variation, thought to grow and mature rapidly compared with other elasmobranchs (Corro‐Espinosa et al., 2011; Lessa et al., 2009). Even within *Rhizoprionodon* there is marked variation in growth parameters, with *R. terraenovae* thought to have a low rate of intrinsic population growth (Cortés, 1998; Márquez‐Farias & Castillo‐Geniz, 1998) compared with *R. taylori* (Simpfendorfer, 1999). Until recently, detailed information regarding the growth functions of sharpnose sharks was restricted to only three of the seven extant species (Corro‐Espinosa et al., 2011), and initial studies in *R. longurio* focused exclusively on the size distribution of individuals and length‐weight relationships (Márquez‐Farias et al., 2005). *R. longurio* is now thought to reach a maximum length of approximately 170 cm (Alatorre‐Ramirez et al., 2013). Recent work has presented evidence that the species exhibits allometric growth in several key morphological structures, likely to have evolved as a result of ontogenetic niche shifts in diet and habitat use (Gayford et al., 2023). The most recent estimates of mean length at maturity are 100.61 and 92.9 cm for males and females, respectively—the highest values reported for any *Rhizoprionodon* species (Corro‐Espinosa et al., 2011). Observations of males maturing at greater length than females in *R. longurio* are particularly intriguing given that this is a viviparous species (Márquez‐Farias et al., 2005). Females typically mature at greater lengths than males in viviparous elasmobranchs, with male‐biased sexual size dimorphism observed predominantly in oviparous taxa (Colonello et al., 2020; Cortés, 2000). Female‐biased sexual size dimorphism is thought to be associated with evolutionary constraint between fecundity and female body size (Colonello et al., 2020). As *R. longurio* matures at a larger size than its congeners (Corro‐Espinosa et al., 2011), sex‐specific selection on body size may be relaxed relative to other *Rhizoprionodon* species. Further work is required to elucidate the details of this relationship, and as a viviparous species exhibiting male‐biased sexual size dimorphism, *R. longurio* represents an important data point for such studies.

Anatomical evidence suggests that *R. longurio* utilises the placental viviparity mode of reproduction, as do its congeners (Márquez‐Farias et al., 2005). There is thought to be a single reproductive cycle per year (Corro‐Espinosa et al., 2011), with gestation of approximately 10–12 months (Márquez‐Farias et al., 2005; Mejía Salazar, 2007) and birthing occurring between the months of April and July (Corro‐Espinosa et al., 2011). Litter sizes are thought to range between 1 and 12 embryos, with an average 7.4 embryos per litter (Márquez‐Farias et al., 2005; Mejía Salazar, 2007). This is much higher than fecundity estimates for other *Rhizoprionodon* species (Capapé et al., 2006; Carlson & Baremore, 2003), and likely the major factor contributing to the resilience of the species to overfishing (Corro‐Espinosa et al., 2011; Furlong‐Estrada et al., 2015). No relationship between litter size and maternal size is known (Mejía Salazar, 2007), lending further credence to the absence of strong selection on female size at maturity. There does however appear to be substantial variation in embryo size within single litters (Márquez‐Farias et al., 2005) as observed in other shark species (Schmidt et al., 2010). The genetic mating system of *R. longurio* still remains unknown and to date no studies have investigated the potential for polyandry or genetic monogamy in any *Rhizoprionodon* species. While not the only hypothesis (Braccini et al., 2007), multiple paternity or sperm storage could explain variations in embryo size and development (Pratt, 1993) in this species. Toxicology studies have found that macronutrients, essential trace elements and non‐essential trace elements are all transferred placentally from mother to embryos in *R. longurio* (Baró‐Camarasa et al., 2023; Frías‐Espericueta et al., 2014). Importantly, the maternal transfer of nutrients is not identical for all elements, with some appearing in greater concentration in maternal tissues, and others in embryonic tissues (Baró‐Camarasa et al., 2023; Frías‐Espericueta et al., 2014). This could result from offloading of maternal toxins (Baró‐Camarasa et al., 2023; Lyons et al., 2013; Mull et al., 2013), differential biochemical requirements of embryos and adults (Green, 2008; Swain & Nayak, 2009) or simply as a result of differences in the chemical properties different elements. Further studies are needed to establish the biochemical details of nutrient transfer in *R. longurio* and the effects these may have on embryonic development and post‐partum ontogeny.

## 5 TROPHIC ECOLOGY

Like the majority of carcharhiniform sharks, *R. longurio* is thought to be a tertiary predator (Alatorre‐Ramirez et al., 2013), predominantly feeding upon teleost fishes, crustaceans and cephalopods (Alatorre‐Ramirez et al., 2013; Márquez‐Farias et al., 2005; Osuna‐Peralta et al., 2014). This diet is similar to that of other *Rhizoprionodon* species (Ba et al., 2013; Delorenzo et al., 2015; Simpfendorfer, 1998); however, the relative contribution of each major prey category appears highly variable, likely due to geographical and seasonal variations in prey abundance (Alatorre‐Ramirez et al., 2013). Such variation is also observed in other *Rhizoprionodon* species, which are typically considered opportunistic predators (Delorenzo et al., 2015; Higgs et al., 2013; Sen et al., 2018). *R. terraenovae* has even been observed foraging on carrion such as the flesh of deceased sea turtles (Delorenzo et al., 2015). The trophic niche breadth of *R. longurio* is less certain, as studies have referred to it as both a specialist and opportunistic predator (Alatorre‐Ramirez et al., 2013; Márquez‐Farias et al., 2005; Osuna‐Peralta et al., 2014). It appears that the species is likely influenced by seasonal variations in prey abundance (Alatorre‐Ramirez et al., 2013), with *R. longurio* potentially specialising on different prey types during different seasons. While *R. longurio* primarily consumes benthic prey (Alatorre‐Ramirez et al., 2013; Márquez‐Farias et al., 2005), pelagic prey species occur in their diet (Alatorre‐Ramirez et al., 2013; Osuna‐Peralta et al., 2014), and thus it has been hypothesised that the species makes vertical migrations through the water column to forage (Alatorre‐Ramirez et al., 2013). Such movements may be more frequent in adults, with juveniles rarely targeting pelagic species (Alatorre‐Ramirez et al., 2013). Other *Rhizoprionodon* species such as *R. taylori* have been found to feed predominantly on pelagic species (Salini et al., 1998; Simpfendorfer, 1998), although even in this species the proportions of pelagic and benthic prey appear to vary geographically (Munroe et al., 2015). There is some evidence for ontogenetic shifts in diet in *R. longurio* (Alatorre‐Ramirez et al., 2013; Trejo Ramírez, 2017), although this is not consistent between all studies (Osuna‐Peralta et al., 2014) and to date no evidence has been found to support sex‐based differences in trophic ecology (Alatorre‐Ramirez et al., 2013). Given apparent seasonal and geographical variation in trophic ecology, long‐term trophic studies will be required to determine the extent to which ontogenetic trophic niche shifts are present. Such shifts have been documented in other *Rhizoprionodon* species (Bethea et al., 2006; Bornatowski et al., 2012) and it would not be surprising if similar trends occurred in *R. longurio*. Importantly, existing studies of trophic ecology in *R. longurio* are restricted to a very small proportion of its total range, predominantly due to lack of research effort. Considering the notable geographic variation in *Rhizoprionodon* trophic ecology, additional studies focussing on populations in the Southern half of *R. longurio*'s range would provide the baseline of knowledge required to truly understand the trophic ecology of this species.

## 6 BEHAVIOURAL AND SENSORY ECOLOGY

While several recently published papers have addressed the trophic, spatial and reproductive ecology of *R. longurio* (Alatorre‐Ramirez et al., 2013; Baró‐Camarasa et al., 2023; Corro‐Espinosa et al., 2011; Frías‐Espericueta et al., 2014; Mejía Salazar, 2007), other areas such as behavioural and sensory ecology have been entirely neglected. No studies have been conducted on these topics, and *R. longurio* is not commonly observed at any SCUBA or ecotourism sites, limiting potential to conduct long‐term behavioural studies. Only a handful of studies exist regarding the sensory capabilities of any *Rhizoprionodon* species (Casper & Mann, 2009; Laforest et al., 2020), and there is no literature addressing fine‐scale behavioural ecology of any members of the genus. Such studies have been conducted both in wild and laboratory‐based populations of several small‐bodied coastal shark species (Gruber & Myrberg Jr, 1977; Jordan et al., 2011; Stroud et al., 2014), and are crucial as they provide invaluable baseline data regarding how sharks interact with both the biotic and abiotic components of their external environment. There are currently no laboratory‐held populations of *R. longurio*; however, non‐experimental approaches focussing on tagging and accelerometer data (as per Watanabe et al., 2019) would be easy to carry out with this species. Behavioural and sensory ecology remain understudied in the majority of elasmobranch taxa (Hueter et al., 2004) and thus in addition to providing key ecological information regarding *R. longurio* itself, such studies would help improve our understanding of taxonomic variation in elasmobranch sensory capabilities and behaviour.

## 7 CONCLUSIONS

Researchers face a race against time to understand the biology and ecology of declining elasmobranch populations before they disappear entirely. *R. longurio* is a commercially significant species (Furlong‐Estrada et al., 2015; Márquez‐Farias et al., 2005) which also fulfils important ecological functions as a tertiary consumer (Alatorre‐Ramirez et al., 2013). Several studies have discussed aspects of the ecology of *R. longurio* (Alatorre‐Ramirez et al., 2013; Baró‐Camarasa et al., 2023; Corro‐Espinosa et al., 2011; Frías‐Espericueta et al., 2014; Mejía Salazar, 2007; Trejo Ramírez, 2017); however, major gaps remain, particularly in the case of behavioural and sensory ecology. Deciphering the nature of the species' migratory behaviour should also be a key focus of future studies. All of these knowledge gaps can be reduced using existing technologies and methodologies that have previously been applied to other shark species. In the case of spatial ecology, acoustic/satellite tagging studies covering both sexes and both adults and juveniles would be sufficient to unravel the extent to which migratory behaviour is observed. In the case of behavioural/sensory ecology a more experimental approach may be required but given the small body size and coastal nature of this species it may well be amenable to temporary captivity. Crucially, existing studies are drawn from only a handful of populations, cumulatively covering a small percentage of the species' total range, and thus additional studies are needed to determine the extent to which *R. longurio* ecology is population‐specific. Each of the future directions outlined here will not only improve our understanding of *R. longurio* and Rhizoprionodon ecology but provide a valuable contribution to our understanding of taxonomic variation in key ecological characteristics of cartilaginous fishes.

## AUTHOR CONTRIBUTIONS

**Joel H. Gayford:** Conceptualization (lead); writing – original draft (lead); writing – review and editing (lead). **Darren A. Whitehead:** Conceptualization (supporting); writing – original draft (supporting); writing – review and editing (supporting).

## FUNDING INFORMATION

The author declares no funding sources for this study.

## CONFLICT OF INTEREST STATEMENT

The author declares no competing interests relevant to this study.

## Data Availability

No data were utilised or generated at any time during the conception or production of this manuscript.
